# Integrative multi-omic profiling of the chronically hypoxic heart: focus on m^6^A and m^6^Am epitranscriptomic regulation

**DOI:** 10.3389/fcell.2026.1756287

**Published:** 2026-01-28

**Authors:** Marketa Hlavackova, Daniel Benak, Dita Sotakova-Kasparova, Kristyna Holzerova, Anton Skriba, Anna Simonova, Hana Cahova, Tatyana Kobets, Tereza Jancova, Frantisek Kolar

**Affiliations:** 1 Laboratory of Developmental Cardiology, Institute of Physiology of the Czech Academy of Sciences, Prague, Czechia; 2 Institute of Organic Chemistry and Biochemistry of the Czech Academy of Sciences, Prague, Czechia; 3 Laboratory of Metabolomics, Institute of Physiology of the Czech Academy of Sciences, Prague, Czechia; 4 Second Faculty of Medicine, Charles University, Prague, Czechia

**Keywords:** epitranscriptomics, heart, hypoxia, m6A, m6Am

## Abstract

Reduced oxygen availability is an environmental factor characteristic of high-altitude conditions that plays a critical role in shaping cellular homeostasis and epigenomic regulation. Adaptation to various models of chronic hypoxia represents a well-recognized physiological process that enhances cardiac tolerance to ischemic stress; however, the molecular mechanisms coordinating metabolic, proteomic, and post-transcriptional remodeling in this adaptive response to low-oxygen conditions remain insufficiently understood. Here, we combined quantitative metabolomic, lipidomic, and proteomic profiling with targeted protein analyses to characterize the molecular landscape of rat hearts adapted to continuous normobaric hypoxia (CNH, 10% O_2_ for 3 weeks). Multi-omics integration revealed tightly coupled remodeling across metabolic and structural domains, consistent with enhanced energetic efficiency and oxidative stress resistance. Pathway enrichment identified coordinated activation of energy reprogramming (AMPK, glycolysis, and PPAR signaling), reinforcement of antioxidant defense (glutathione metabolism), membrane remodeling (glycerophospholipid and peroxisomal pathways), and protein quality control (autophagy–lysosome and proteasome systems). Beyond these canonical adaptive responses, CNH markedly affected the epitranscriptomic machinery: both m^6^A demethylases ALKBH5 and FTO – enzymes previously linked to cardioprotective effects – were upregulated, accompanied by increased abundance of multiple m^6^A readers (YTHDF1–3, YTHDC1), whereas methyltransferases METTL3 and PCIF1 remained stable. At the level of RNA modifications, global m^6^A levels in total RNA were unchanged, whereas m^6^Am levels were significantly increased under hypoxia. These results demonstrate that chronic hypoxia reprograms the heart not only at the metabolic and proteomic levels but also through epitranscriptomic regulation, suggesting that RNA methylation dynamics may contribute to the cardioprotective phenotype. Collectively, our findings provide a system-level framework linking metabolic flexibility, redox balance, and post-transcriptional control during hypoxic adaptation.

## 1 Introduction

Adaptation to chronic hypoxia represents a quintessential example of how environmental factors can reprogram cellular and molecular networks to enhance physiological resistance to acute hypoxia. In the heart, sustained exposure to low oxygen levels elicits a well-established adaptive response that increases myocardial tolerance to acute ischemic injury (Kolar and Ostadal, 2004). This phenomenon, first recognized in the 1960s in populations living at high altitudes under chronically low oxygen conditions, has long intrigued both basic scientists and clinicians because of its potential to mitigate the burden of ischemic heart disease (IHD) (Hurtado, 1960). Despite decades of research, however, the direct clinical application of hypoxia-based interventions remains limited. The major barrier lies in the unpredictable nature of acute ischemic events, which often preclude preconditioning strategies based on controlled hypoxic exposure. Nevertheless, elucidating the cellular and molecular mechanisms underlying hypoxia-induced cardioprotection remains of paramount importance. By identifying the signaling networks and molecular effectors that mediate this endogenous defense, we may uncover pharmacologically targetable pathways that could be exploited prophylactically – or even acutely – offering more practical therapeutic strategies than hypoxic preconditioning itself.

At the molecular level, chronic hypoxia activates a coordinated transcriptional response primarily mediated by hypoxia-inducible factors (HIFs), which regulate processes such as erythropoiesis, angiogenesis, and metabolic reprogramming from oxidative phosphorylation to glycolysis, through transcriptional control of more than 1,000 target genes (Seagroves et al., 2001; Benak et al., 2025). Beyond HIFs, several other molecular mediators – including ATP-sensitive potassium channels, reactive oxygen species, nitric oxide, and a variety of kinases (e.g., protein kinase C, mitogen-activated protein kinases) – have been implicated in hypoxia-induced cardioprotection (Kolar and Ostadal, 2004; Chytilova et al., 2015; Hlavackova et al., 2010; Hlavackova et al., 2007; Micova et al., 2016; Alanova et al., 2017). However, despite these advances, our current understanding of this complex adaptive response remains fragmented.

Recent advances in multi-omics technologies have made it possible to interrogate this complexity directly. Integrating proteomic, metabolomic, and lipidomic data enables the discovery of coordinated networks underlying energetic reprogramming, membrane remodeling, and stress adaptation in the heart. However, some key regulatory processes – particularly those involving epitranscriptomic modifications – remain difficult to capture by standard proteomic workflows. These modifications, such as N^6^-methyladenosine (m^6^A) and N^6^,2′-O-dimethyladenosine (m^6^Am), modulate RNA stability, splicing, and translation, and are controlled by distinct sets of “writers”, “erasers”, and “readers” (Kumar and Mohapatra, 2021; Benak et al., 2024a; Hlavackova et al., 2025; Benak et al., 2026; Benak et al., 2023; Oerum et al., 2021). Accumulating evidence indicates that hypoxia influences both the deposition and removal of these RNA marks, while HIF-1α itself is regulated by m^6^A-dependent mechanisms (Benak et al., 2025). Yet, the contribution of m^6^A/m^6^Am machinery to cardiac hypoxic adaptation remains poorly defined, partly because the low abundance of these regulators often limits their detection in discovery-based proteomics.

To address these knowledge gaps, the present study integrates proteomic, metabolomic, and lipidomic profiling of chronically hypoxic hearts to construct a system-level view of cardiac adaptation. Recognizing the underrepresentation of epitranscriptomic regulators in global proteomic datasets, we complemented this analysis with targeted Western blot quantification of major m^6^A/m^6^Am writers, erasers, and readers. Together, these complementary approaches aim to delineate the molecular landscape of chronic hypoxia–induced cardioprotection and identify candidate regulatory mechanisms that bridge metabolic adaptation and post-transcriptional control.

## 2 Materials and methods

Unless stated otherwise, all chemicals were obtained from Sigma-Aldrich (St. Louis, United States).

### 2.1 Animals and adaptation to chronic hypoxia

Adult (3–4 months old) male Sprague–Dawley rats (Center of Experimental Medicine of the Institute for Clinical and Experimental Medicine, Prague) were assigned to either continuous normobaric hypoxia (CNH; n = 4) or normoxic control conditions (n = 6). CNH animals were exposed to 10% O_2_ in a normobaric chamber equipped with hypoxic generators (Everest Summit, Hypoxico, NY, United States) for 3 weeks, representing an established model of cardioprotective adaptation (Neckar et al., 2017). This model reproduces sustained systemic hypoxemia characteristic of high-altitude conditions in humans while avoiding confounding effects associated with changes in barometric pressure. No reoxygenation occurred during this period. Control rats were maintained at room air for the same duration. All animals were housed under controlled environmental conditions (23 °C; 12 h:12 h light–dark cycle; lights on at 6:00 a.m.) with free access to water and standard chow.

The animals for analysis were deeply anesthetized with pentobarbital (50 mg/kg) and killed by cervical dislocation immediately after the cessation of hypoxic exposure. Intact hypoxic and normoxic hearts were rapidly excised and washed in a cold (0 °C) saline. Hearts were dissected into the right ventricle (RV), the free wall of the left ventricle (LV), and the septum (Balkova et al., 2009). All collected tissue segments were weighed, frozen, and stored in liquid nitrogen until use. Experiments were performed in accordance with a valid experimental project, Permit No. 66/2021. The project is approved by the Animal Care and Use Committee of the Institute of Physiology of the Czech Academy of Sciences, as well as by the Resort Professional Commission of the CAS for Approval of Projects of Experiments on Animals. The animals were housed in facilities accredited by the Czech Ministry of Agriculture. Experiments were carried out under veterinary supervision, complying with Act No. 246/1992 Coll. and Decree No. 419/2012 Coll., implementing Directive 2010/63/EU of the European Parliament and of the Council regarding the protection of animals used for scientific purposes. The 3Rs principles were applied to the maximum extent possible.

### 2.2 Metabolomic and lipidomic analysis

Metabolomic and lipidomic analysis was performed to investigate the effects of CNH on LV tissue samples. Tissue samples were prepared following established protocols (Benak et al., 2024b; Sistilli et al., 2021; Tsugawa et al., 2020). For extraction, a biphasic solvent system of cold methanol, methyl *tert*-butyl ether (MTBE), and water (Cajka et al., 2017; Cajka et al., 2023) was used. Four different LC-MS platforms were used for metabolomics and lipidomics profiling: (i) metabolomics of polar metabolites in positive ion mode (BEH Amide platform), (ii) metabolomics of polar metabolites in negative ion mode (HSS T3 platform), (iii) lipidomics in positive ion mode (BEH C18 platform), (iv) lipidomics in negative ion mode (BEH C18 platform). List of annotated complex lipids and polar metabolites is in Supplementary Table S1.

Internal standards were added to the extraction and resuspension solvents and monitored throughout the analytical workflow to assess extraction efficiency and instrument stability. Sample injection order was randomized. Pooled quality control (QC) samples, prepared from aliquots of all LV extracts, were injected periodically throughout each LC–MS platform sequence. Procedural blanks were analyzed to monitor background contamination. Instrument performance and signal stability were evaluated based on internal standard responses, retention time consistency, and signal reproducibility across QC injections.

### 2.3 Proteomic analysis

Proteomic analysis was performed to investigate the effects of CNH on LV tissue. The samples were homogenized and peptides prepared according to established protocols (Benak et al., 2024c). In short, peptide mixtures were analyzed by liquid chromatography coupled with high-resolution mass spectrometry (Orbitrap Exploris 480 with FAIMS interface) using a data-independent acquisition (DIA) strategy. Peptides were separated on a C18 column with a 120-min gradient, and DIA settings included an MS1 scan range of m/z 350–1,500 at 60,000 resolutions, followed by staggered MS2 windows with high-energy collision dissociation. Acquired DIA datasets were processed in Spectronaut software, and proteins were quantified based on relative peptide abundance. Comparisons were made between CNH-adapted and normoxic LV samples.

Western blot analysis was employed to validate key findings from the proteomic study as described previously (Benak et al., 2024c). Significant upregulation of carbonic anhydrase 2 (CA2) and 15-lipoxygenase 1 (ALOX15) was confirmed in LV samples (Supplementary Figure S1), corroborating the proteomic data. These proteins were selected as representative targets based on their consistent regulation in the discovery dataset, established relevance to hypoxia-related metabolic and redox pathways, and the availability of well-validated antibodies suitable for quantitative immunoblotting.

### 2.4 Multi-omics data integration

To integrate proteomic, metabolomic, and lipidomic datasets and identify discriminating molecular features between experimental groups, we applied the DIABLO framework (Data Integration Analysis for Biomarker discovery using Latent cOmponents (Singh et al., 2019)) implemented in the mixOmics R package (Rohart et al., 2017). Features containing missing values were removed prior to analysis. Because the proteomic dataset was the largest (4,530 quantified proteins), the top 500 most variable proteins were retained to ensure balanced contribution across datasets. All detected metabolites (n = 102) and lipids (n = 479) were included. A multi-block partial least squares discriminant analysis (PLS-DA) was performed with parameters optimized for the present data. From each dataset, the 20 features with the highest loadings on each component were selected for downstream interpretation. Loadings were extracted for the first two components (Supplementary Figure S2; Supplementary Table S2). To explore cross-omics relationships among top discriminating variables, sparse generalised canonical correlation discriminant analysis (sgccda) was used (Figure 3D; Supplementary Table S3).

### 2.5 Tissue processing and western blotting analysis

As described earlier, tissue homogenization, protein separation (Semenovykh et al., 2022), and immunodetection (Holzerova et al., 2015) were performed. Frozen LV myocardium was pulverized in liquid nitrogen to a fine powder followed by Potter-Elvehjem homogenization in eight volumes of homogenization buffer [12.5 mM TRIS, 2.5 mM EGTA, 1 mM EDTA, 250 mM sucrose, 6 mM 2-mercaptoethanol, protease inhibitor cocktail (Roche Diagnostics, Germany) and phosphatase inhibitor cocktail (Roche Diagnostics, Germany), pH 7.4]. The protein concentration in homogenates was measured by the Bradford method (Bio-Rad, United States). The LV homogenates were subjected to SDS electrophoresis on 10% polyacrylamide gels (Mini-PROTEAN TetraCell; Bio-Rad, United States) and electrotransferred onto PVDF membranes (0.2 μm pore size; Bio-Rad, United States). Subsequently, membranes were blocked with 5% blotting-grade blocker (Bio-Rad, United States) in PBS containing Tween 20 (1%) for 1 h and incubated with appropriate primary and secondary antibodies (diluted in 1% blotting-grade blocker and 1% Tween 20 in PBS): anti-ALKBH5 (Abcam, United Kingdom; ab195377, 1:1,400, overnight), anti-FTO (Abcam, United Kingdom; ab92821, 1:1,000, overnight), anti-METTL3 (Abcam, United Kingdom; ab195352, 1:1,000, overnight), anti-METTL4 (Invitrogen, United States; PA5-97202, 1:1,400, overnight), anti-PCIF1 (Invitrogen, United States; PA5-110081, 1:1,400, overnight), anti-YTHDC1 (Abcam, United Kingdom; ab220159, 1:1,400, overnight), anti-YTHDC2 (Abcam, United Kingdom; ab220160, 1:1,400, overnight), anti-YTHDF1 (Abcam, United Kingdom; ab157542, 1:1,400, overnight), anti-YTHDF2 (Invitrogen, United States; PA5-70853, 1:1,400, overnight), anti-YTHDF3 (SAB21022736, 1:1,400, overnight), anti-mouse secondary antibody (Invitrogen, United States; 31432, 1:10,000, 1 h) and anti-rabbit secondary antibody (Bio-Rad, United States; 170–6515, 1:10,000, 1 h). The same amount of protein was loaded on the gels and all samples from all experimental groups were always analyzed on the same membrane. The same reference sample was run on each gel and was used for normalization. At the same time, results were recalculated to the total protein amount gained by Ponceau S staining (Semenovykh et al., 2022), a method preferred over the use of housekeeping proteins as loading controls (Moritz, 2017). Each biological sample was analyzed in at least three technical replicates, which were averaged to yield a single value per animal, and statistical analyses were performed using biological replicates. The membranes were visualized by enhanced chemiluminescence (ECL) substrates (SuperSignal™ West Dura Extended Duration Substrate or SuperSignal™ West Femto Maximum Sensitivity Substrate, Thermo Scientific, United States) using a ChemiDoc™ system (Bio-Rad, United States).

### 2.6 m^6^A/m^6^Am quantification in left ventricle tissue

According to the manufacturer’s instructions, total RNA was isolated from control and CNH adapted LVs with RNAzol® RT.

RNA samples (500–1,000 ng) were digested by 1 U of Nuclease P1 (M0660S, New England BioLabs, United States) in 50 mM ammonium acetate buffer (pH 5.5) and 0.3 U of snake venom phosphodiesterase (P3243, Merck, Germany) at 37 °C for 2 h. After that, the pH of the solution was adjusted to ≈8 (by addition of 1.5 ekv. of ammonium acetate pH 9.2) and all nucleotides were dephosphorylated by 0.01 U of Shrimp Alkaline Phosphatase (M0371S, New England BioLabs, United States) at 37 °C for 1 h. At this point reaction was spiked with the mixture of isotopically labeled D3-m^6^A (TRC-M275897, LGC, United States) and D3-m^6^Am (TRC-D447417, LGC, United States) standards (10 mM final concentration). Digested samples were filtered with Vivacon 500, 10 kDa centrifugal filters (VN01H02, Sartorius, Germany). The flow-through was transferred to the HPLC vial for subsequent LC-MS analysis.

The separation of the digested RNA samples was performed by HPLC system (Acquity Premier, Waters, United States) on a C18 column (Acquity Premier HSS T3, 1.8 µm, 2.1 × 100 mm, Waters, United States) at 35 °C using a gradient of water (A) and acetonitrile (B), each containing 0.1% (v/v) formic acid. The gradient was 0–2 min, 100% A; 8 min, 85% A; 8.5 min, 10% A; 11 min, 10% A; 11.1 min, 100% A; 17 min, 100% A. The flow rate was 0.25 mL/min. The autosampler was running at 10 °C and injection volume was 5 µL. The HPLC system was coupled online to a triple quadrupole mass spectrometer (Xevo TQ Absolute, Waters, United States). The source settings were: capillary voltage 3 kV, source temperature 150 °C, cone voltage 20 V, desolvation temperature 600 °C, desolvation gas flow, 600 L/h, positive ion mode. The instrument was operated in MRM (multiple reaction monitoring) mode with 0.024 s dwell time and following transitions: 282.075 > 150.03 (m^6^A), 285.138 > 153.029 (D3-m^6^A), 296.075 > 149.977 (m^6^Am), 299.138 > 153.010 (D3-m^6^Am).

Data analysis was conducted using TargetLynx XS V4.2 software. Quantification was achieved by measuring the MRM signal peak area (area under the curve, AUC) and applying single-point calibration based on the known value of a spiked isotopically labeled standards (Supplementary Figure S3).

### 2.7 Data processing and statistical analyses

For proteomic, metabolomic, and lipidomic analyses, all available biological replicates were used, comprising normoxic controls (n = 6) and CNH-adapted animals (n = 4). For immunochemical analyses, a subset of animals was used (n = 4 per group), as specified in the corresponding figure legends.

For metabolomic and lipidomic datasets, data were normalized using mTIC (total ion chromatogram of all annotated metabolites), log_10_-transformed, and Pareto-scaled prior to statistical evaluation. Group comparisons were performed with unpaired two-tailed t-tests in the R environment (version 4.3.2). Metabolites were evaluated based on fold-change and p-value, and ranked according to variable importance in projection (VIP) scores from PLS-DA.

For proteomic datasets, raw files were processed in Spectronaut 14. Differential expression analysis was conducted in R (version 4.5.0) using the MSstats package (version 4.116.1) (Choi et al., 2014; Kohler et al., 2023). Data were normalized using a median-based approach and summarized by Tukey’s polish method. Proteins represented by only a single peptide were excluded. Missing values were imputed using the MSstats algorithm, except where entire proteins were absent across runs (Kohler et al., 2023). Proteins were considered differentially expressed at |log_2_ fold change| ≥ 0.5 and p ≤ 0.05. Functional annotation of significant proteins was performed by over-representation analysis (ORA) against the KEGG database (Kanehisa, 2019; Kanehisa et al., 2025; Kanehisa and Goto, 2000) (https://www.genome.jp/).

For immunochemistry experiments, statistical analyses were performed in GraphPad Prism 8 (GraphPad Software, United States). Data are presented as mean ± SD or, where indicated, as median with interquartile range (box) and minimum–maximum values (whiskers). Between-group comparisons were performed using unpaired two-tailed Student’s t-tests. Differences were considered significant at p ≤ 0.05. The number of biological replicates (n) used for each analysis is indicated in the corresponding figure and table legends.

## 3 Results

### 3.1 Multi-omic analysis of the chronically hypoxic heart

To get a better understanding of the effect of CNH on the heart, proteomic, metabolomic, and lipidomic analysis were performed.

**FIGURE 1:**
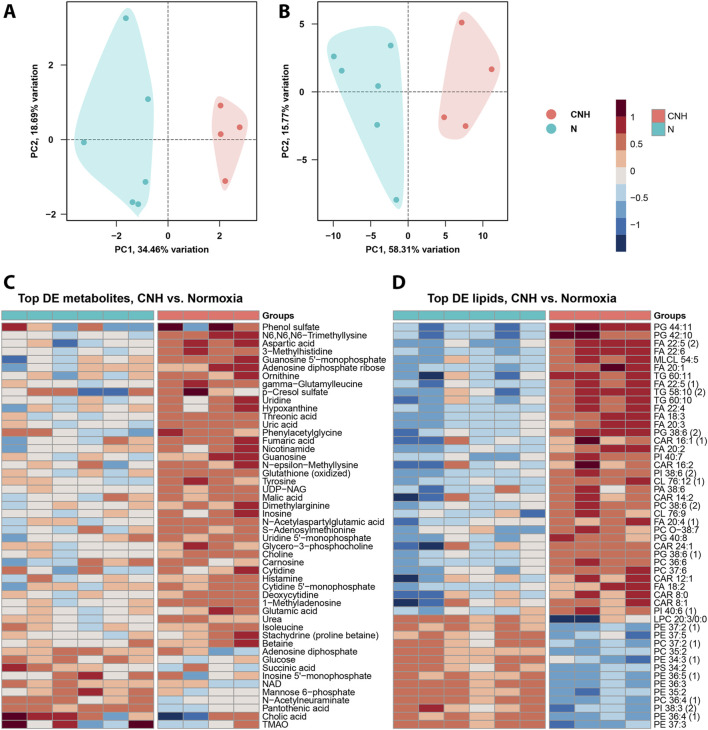
Metabolomics and lipidomics analyses in left ventricular samples between continuous normobaric hypoxia (CNH) and normoxic (N) groups. Principal component analysis (PCA) of the detected metabolites **(A)** and lipids **(B)** indicated high discrimination between the experimental groups. **(C)** Top DE metabolites selected by log2FC (Supplementary Table S1). **(D)** Top DE lipids selected by log2FC (Supplementary Table S1).

Metabolomic and lipidomic analyses revealed significant differences in cardiac metabolite and lipid profiles between the experimental groups. PCA demonstrated clear separation between groups, indicating substantial metabolic and lipidomic reprogramming under CNH (34% and 58% of explained variance, respectively; Figures 1A,B). DE analysis identified significant changes in 37 metabolites (Figure 1C) and 250 lipids (Figure 1D). These changes involved metabolites and lipids related to energy metabolism (e.g., glucose-6-phosphate, succinic acid) and membrane remodeling (e.g., phosphatidylglycerols and fatty acids), underscoring the metabolic and structural hypoxic adaptations. These results are summarized in Supplementary Table S4.

**FIGURE 2:**
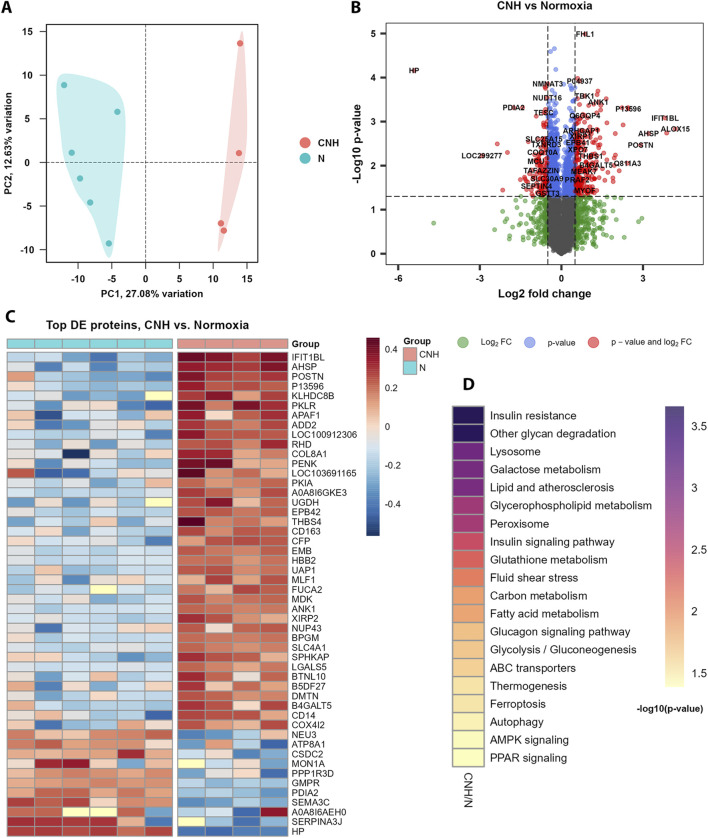
Differential expression of proteins in left ventricular samples between continuous normobaric hypoxia (CNH) and normoxic (N) groups. **(A)** PCA of the expressed proteins showed clear discrimination between the groups (27% of explained variance). **(B)** Results of the differential expression (DE) anaysis; red dots indicate proteins that met criteria of significance (Supplementary Table S6). **(C)** Top DE proteins selected by log2FC (Supplementary Table S7). **(D)** Functional annotation of the results of DE analysis using KEGG database of pathways (Supplementary Table S8).

Proteomic analysis revealed distinct cardiac protein expression profiles between the CNH and normoxic groups (Supplementary Tables S5-S6). Principal component analysis (PCA) showed clear separation between groups, indicating substantial proteomic reprogramming under CNH (27% of explained variance; Figure 2A). Analysis of the top differentially expressed (DE) proteins revealed 129 downregulated and 237 upregulated proteins (Supplementary Table S7; Figures 2B,C). Pathway enrichment in CNH hearts (Supplementary Table S8; Figure 2D) indicated regulation of metabolic and stress-response networks. Affected pathways converged on energetic reprogramming (AMPK signaling; glycolysis/gluconeogenesis), lipid/PPAR-associated metabolic flexibility (fatty acid metabolism, peroxisome, glycerophospholipid metabolism), antioxidant systems (glutathione metabolism), and cellular quality control mechanisms (autophagy/lysosome), along with pathways linked to ferroptosis.

**FIGURE 3:**
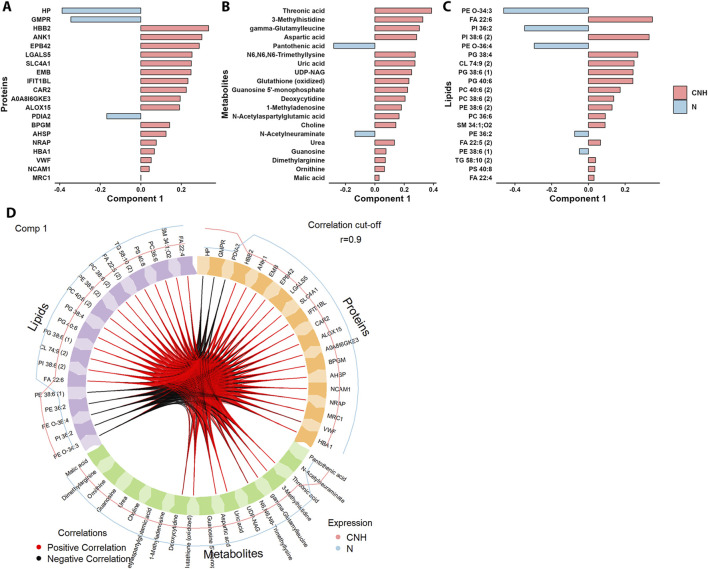
Multi-omics integration of proteins, metabolites and lipids in left ventricular samples. **(A)** Top proteins discriminating between the experimental groups from the block PLS-DA. Values of each loading vector indicated the importance of a variable in a component. **(B)** Metabolites and **(C)** top lipids with the highest contribution to the variation between the tested conditions. **(D)** Circos plot showing mutual correlations among the top discriminating proteins, metabolites and lipids (Supplementary Table S3).

To integrate proteomic, metabolomic, and lipidomic alterations and assess their coordinated regulation under CNH, a multi-omics analysis was performed using the DIABLO framework. Despite the simple two-group design, this approach confirmed a high degree of cross-omics concordance (average inter-block correlation > 0.98; Supplementary Figure S2A), indicating that proteomic, metabolomic, and lipidomic responses were tightly coupled. Component 1 of the multi-block PLS-DA clearly separated CNH from normoxic hearts (Supplementary Figure S2B) and explained 26%, 34%, and 47% of the total variance in proteomic, metabolomic, and lipidomic datasets, respectively, while Component 2 captured within-group variability. The features contributing most to Component 1 (top 20 per dataset; Figures 3A–C; Supplementary Table S2) defined a coherent biological module rather than independent changes in each omic layer. Proteins with the highest loadings were predominantly associated with hematopoiesis and oxygen transport (GO:0030097) – including HBA1, HBB2, ANK1, SPTA1, SLC4A1, EPB42, BPGM, and HP – and with immune-system processes (GO:0002376) represented by MRC1, LGALS9, and CD1D1. These protein changes were positively correlated with metabolites involved in amino-acid and redox metabolism (e.g., glutathione, aspartate, pantothenic acid) and with membrane lipids such as phosphatidylethanolamines (PE), phosphatidylinositols (PI), and cardiolipins (CL), which participate in mitochondrial and membrane remodeling. Together, the DIABLO analysis quantitatively confirmed that CNH induces a coordinated, cross-omics adaptation encompassing erythrocyte/oxygen transport proteins, metabolic intermediates, and structural lipids. This integrated remodeling reflects the systemic and cardiac adjustments that support energy homeostasis and adaptation to chronic hypoxia (Figure 3D; Supplementary Table S3).

### 3.2 Effect of chronic hypoxia on epitranscriptomic regulations

**FIGURE 4:**
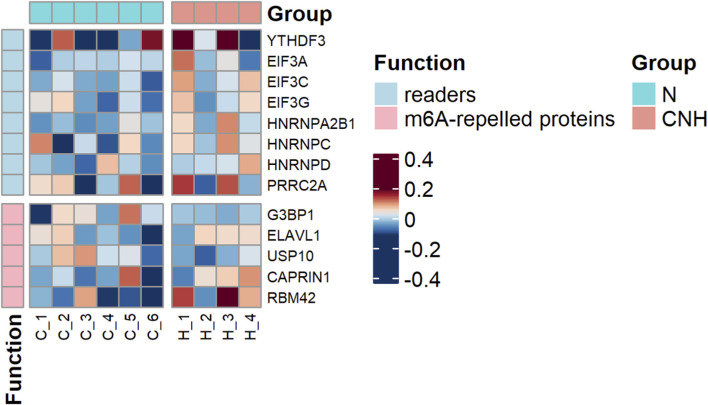
Proteomic profiling of m^6^A regulatory proteins in left ventricular samples between continuous normobaric hypoxia (CNH) and normoxic (N) groups. Heatmap shows relative abundance of m^6^A regulators (readers and m^6^A-repelled proteins) detected by quantitative proteomic analysis. Color scale represents normalized expression changes (log2FC). Due to the low detection of core m^6^A regulatory machinery (writers and erasers) in the proteomic dataset, ten key regulators were further quantified by immunoblotting (see Figure 5).

**FIGURE 5:**
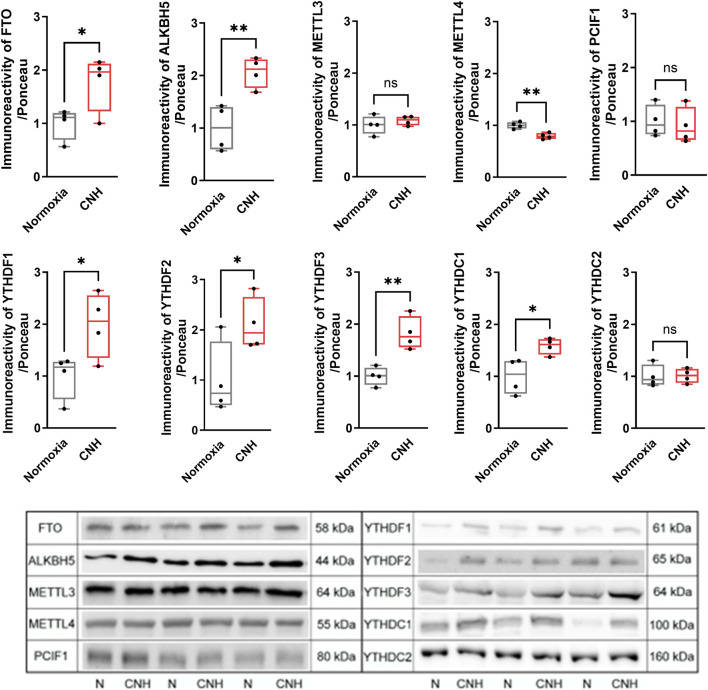
Effect of continuous normobaric hypoxia (CNH) adaptation on protein levels of m^6^A and m^6^Am regulators in the left ventricular myocardium. Data are presented as medians, with boxes indicating the interquartile range and whiskers representing the minimum and maximum values. Protein levels were normalized to total protein using Ponceau S staining, a method recommended over conventional housekeeping proteins. n = 4; *p < 0.05; **p < 0.01 (t-test). N, normoxia.

To explore whether adaptation to CNH alters the epitranscriptomic machinery in the heart, we first performed proteomic profiling of m^6^A-related proteins. The analysis identified several m^6^A readers (YTHDF3, EIF3A, EIF3C, EIF3G, HNRNPA2B1, HNRNPC, HNRNPD, PRRC2A) and m^6^A-repelled proteins (G3BP1, ELAVL1, CAPRIN1, RBM42); however, none of the observed changes reached the predefined threshold for statistical significance. Moreover, the writers and erasers of these RNA modifications were not represented among the detected proteins (Figure 4). Given the limited proteomic detection of the enzymatic machinery responsible for m^6^A and m^6^Am modification, we next quantified the key regulatory proteins by Western blotting (Figure 5).

CNH strongly upregulated the demethylases ALKBH5 (+106%) and FTO (+77%), whereas methyltransferases METTL3 and PCIF1 remained unchanged and METTL4 decreased (−21%). Among m^6^A readers, all were significantly increased except YTHDC2, which was unaffected: YTHDF1 (+99%), YTHDF2 (+110%), YTHDF3 (+82%), and YTHDC1 (+58%).

**FIGURE 6:**
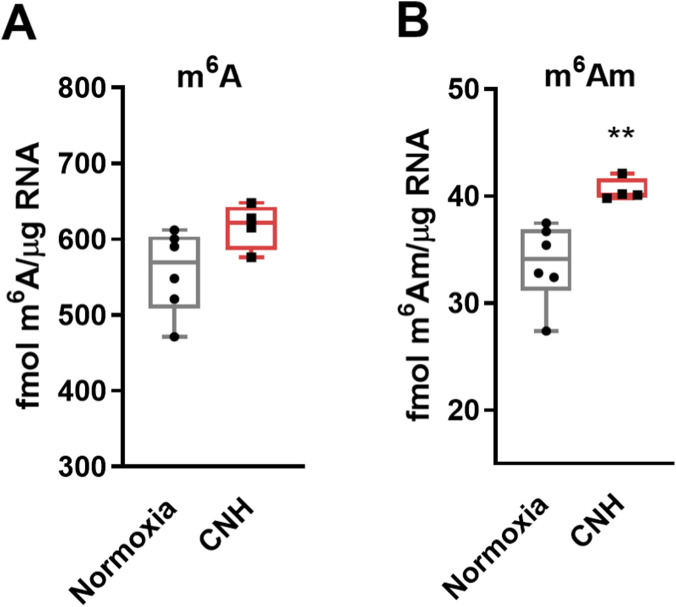
Effect of continuous normobaric hypoxia (CNH) adaptation on m^6^A **(A)** and m^6^Am **(B)** levels in total RNA isolated from the left heart ventricle. Data are presented as medians with boxes and whiskers representing the interquartile range and minimum to maximum values, respectively. n = 4–6; **p < 0.01 (t-test).

Analyses of m^6^A and m^6^Am levels (Figure 6) revealed that in cardiac (LV) total RNA the level of m^6^A was unchanged following CNH, whereas m^6^Am levels increased significantly by approximately 20%.

## 4 Discussion

Our data indicate that (i) CNH induces a coordinated remodeling of the cardiac proteome, metabolome, and lipidome consistent with enhanced metabolic flexibility and stress resistance; (ii) this systemic adaptation engages pathways related to energy reprogramming, antioxidant defense, membrane remodeling, and protein quality control; and (iii) CNH is accompanied by profound reorganization of the m^6^A/m^6^Am epitranscriptomic machinery, characterized by upregulation of the demethylases ALKBH5 and FTO and a concerted increase in several m^6^A reader proteins. Collectively, these findings reveal that chronic hypoxic adaptation recruits not only classical metabolic and structural remodeling but also post-transcriptional regulatory layers that may contribute to the maintenance of cardiac homeostasis and the establishment of a cardioprotective phenotype.

Our multi-omics data highlight extensive metabolic and proteomic remodeling underlying adaptation to chronic hypoxia. Indeed, it has been well documented that prolonged hypoxic exposure activates oxygen-sensitive transcriptional regulators, including HIF-1 and PPARs, which collectively promote a metabolic shift toward greater reliance on glucose utilization as more oxygen-efficient substrate compared to fatty acids. This metabolic reprogramming enhances ATP generation efficiency and preserves contractile function under limited oxygen availability (Essop, 2007). In line with this, we observed coordinated regulation of glycolysis, AMPK, and PPAR signaling pathways, together with modulation of the TCA cycle and fatty acid metabolism, indicating enhanced metabolic flexibility. Interestingly, the HIF-1 signaling pathway itself was not among the KEGG-enriched annotations, likely reflecting the adaptive steady state reached after 3 weeks of CNH exposure, when HIF-1 activity becomes attenuated – as previously reported in other tissues (Chavez et al., 2000).

Beyond the well-established HIF-1–mediated metabolic reprogramming, our proteomic data revealed additional adaptive layers that have received little attention in the context of chronic hypoxia. Several proteins associated with mitochondrial quality control and mitophagy (including PINK1, SQSTM1, and GABARAPL2/3) were altered, indicating that the mitophagy machinery is remodeled during long-term hypoxic adaptation. This observation is consistent with our recent findings in a mouse model of intermittent hypobaric hypoxia, where HIF-1α–dependent mitophagy contributed to cardioprotection (Alanova et al., 2024). The convergence of these findings across distinct experimental paradigms suggests that modulation of mitochondrial quality control represents a common feature of chronic hypoxic adaptation, regardless of species or exposure profile.

Concurrent changes in coenzyme Q biosynthetic enzymes (COQ8A, COQ9) and peroxisomal lipid remodeling and antioxidant factors (CAT, ABCD1/3, PEX11B) further indicate reinforcement of mitochondrial redox capacity (Yuan et al., 2021; Guo et al., 2024). Moreover, modulation of RNA-binding and turnover proteins (CNOT1, RBM12, CPEB4, METTL15, CSDC2) together with components of the hexosamine/O-GlcNAc pathway (GFPT1, UAP1, OGA) points to a coordinated post-transcriptional and post-translational remodeling that complements the epitranscriptomic changes observed (Cairns et al., 2022; Dontaine et al., 2022; Qiu et al., 2024; Shi, 2023). These findings suggest that chronic hypoxia triggers a multilayered adaptive program encompassing not only metabolic and structural pathways but also mitochondrial maintenance and RNA metabolism, thereby contributing to the establishment of a durable cardioprotective phenotype.

Given the changes in RNA metabolism–associated proteins and our focus on epitranscriptomic (m^6^A and m^6^Am) regulation as a post-transcriptional layer capable of fine-tuning cellular adaptation, together with the established interconnection between HIF-1 signaling and the m^6^A pathway (Benak et al., 2025), we next explored epitranscriptomic regulation in cardioprotective hypoxic adaptation.

Among demethylases, ALKBH5 and FTO are 2-oxoglutarate/Fe^2+^−dependent dioxygenases whose enzymatic activity requires molecular oxygen; their function may therefore be attenuated under hypoxia (Benak et al., 2024d). The *ALKBH5* gene is a direct transcriptional target of HIF-1α, and its induction by hypoxia has been reported in multiple cell types (Thalhammer et al., 2011; Wang et al., 2021). Such upregulation may therefore represent a compensatory mechanism to maintain ALKBH5 activity despite reduced oxygen availability. Consistent with this concept, we observed increased ALKBH5 protein levels in hypoxic rat hearts. By contrast, a study in naked mole rats (*Heterocephalus glaber*) exposed to acute hypoxia (7% O_2_, 24 h) reported decreased cardiac ALKBH5 levels (Ingelson-Filpula et al., 2024), which may reflect species-specific adaptations in an organism renowned for extreme hypoxia tolerance (Kadamani et al., 2024). The regulation of FTO under hypoxia appears more complex. Although hypoxia-responsive elements bound by HIF-1α have been identified in the *Fto* promoter, HIF-1 has been suggested to suppress rather than activate its transcription (Wang et al., 2024). Accordingly, reduced FTO levels has been observed in hypoxic cardiomyocytes (Deng et al., 2021) as well as in naked mole rat hearts subjected to acute hypoxia (Ingelson-Filpula et al., 2024). In contrast, our data demonstrate increased cardiac FTO protein levels in rats adapted to chronic hypoxia, suggesting that FTO regulation is highly context-dependent, potentially differing with species, tissue type, and the duration or severity of hypoxic exposure.

Similar to demethylases, other m^6^A/m^6^Am regulators are also responsive to hypoxia under certain conditions, including METTL3 (Wang et al., 2021; Yao et al., 2020; Song et al., 2019; Su et al., 2020; Zhao et al., 2021), METTL4 (Hao et al., 2020), YTHDF1-3 (Wang et al., 2021; Hu et al., 2021), and YTHDC1-2 (Wang et al., 2021), as demonstrated *in vitro* across various cell types. In contrast to ALKBH5 and FTO, which are downstream targets of HIF-1, YTHDC2 has been reported to promote HIF-1α translation (Tanabe et al., 2016). However, *in vivo* studies on the regulation of m^6^A/m^6^Am machinery under hypoxia remain limited. The previously mentioned study (Ingelson-Filpula et al., 2024) in naked mole rats not only reported decreased cardiac ALKBH5 and FTO levels but also observed downregulation of YTHDF1 – finding that also contrasts with our results, where we observed upregulation of this reader. Additionally, while the latter study reported unchanged levels of METTL3 and YTHDF2-3, our findings demonstrated stable METTL3 levels but upregulated YTHDF2-3 in the hypoxic rat heart. Another study (Shi et al., 2019), which focused on the liver and kidneys, described decreased YTHDF1 levels in highland cattle compared to lowlanders, further supporting the notion that m^6^A/m^6^Am regulators may exhibit tissue-specific and species-specific responses to hypoxia.

Importantly, our findings revealed a counterintuitive increase in FTO protein levels accompanied by elevated m^6^Am levels, a relationship not attributable to changes in the known m^6^Am writers PCIF1 or METTL4. Given that FTO functions as a demethylase, one would typically expect its upregulation to result in decreased m^6^Am abundance. However, m^6^Am remains an understudied modification, and its full regulatory network is not yet well defined. For example, while numerous m^6^A-binding reader proteins have been identified, specific readers for m^6^Am have yet to be discovered. It is therefore plausible that additional, as-yet-uncharacterized regulators or cap-binding proteins may influence m^6^Am dynamics independently of FTO activity. Alternatively, because FTO is a dioxygenase that requires molecular oxygen as a cofactor, its enzymatic activity may be intrinsically limited under hypoxic conditions despite its increased expression. Thus, the observed upregulation of FTO may represent an insufficient compensatory response to maintain m^6^Am homeostasis under sustained low-oxygen stress. However, even upregulation of FTO under hypoxic conditions could prove cardioprotective during reperfusion when oxygen levels are increased and its activity is therefore restored. Future studies will be needed to elucidate the precise mechanistic links between FTO expression, its enzymatic activity under different oxygen levels, and the regulation of m^6^Am, as these questions extend beyond the scope of the present work.

Altogether, these results highlight extensive epitranscriptomic regulation in the hearts of rats adapted to chronic hypoxia, a well-established cardioprotective intervention, and suggest a potential regulatory mechanism underlying the hypoxic adaptation. This observation is consistent with other reports demonstrating epitranscriptomic remodeling in various cardioprotective contexts. For instance, we have previously shown that fasting – a potent endogenous cardioprotective stimulus – is also associated with alterations in m^6^A/m^6^Am machinery and decreased methylation levels (Benak et al., 2024b). Notably, in hearts of fasting rats, we observed increased levels of both demethylases ALKBH5 and FTO, consistent with their known protective effects; however, other components of the regulatory network differed, particularly m^6^A readers, which were predominantly upregulated after adaptation to hypoxia but downregulated following fasting. The upregulation of FTO and decrease in m^6^A levels was also observed on mice intermittent fasting model reported by Xu et al. (2022). These similarities and differences underscore the complexity of m^6^A/m^6^Am regulation in the heart under distinct cardioprotective conditions and highlight the need for a more detailed characterization of its role in the establishment of the cardioprotective phenotype. Nevertheless, while epitranscriptomic remodeling is consistently observed across multiple cardioprotective paradigms, including CNH, the present study is not designed to establish causality, and targeted gain- or loss-of-function approaches will be required to directly define the contribution of specific regulators to hypoxia-induced cardioprotection.

Understanding how oxygen availability modulates RNA methylation and metabolic pathways in the heart may open new therapeutic opportunities. Pharmacological agents mimicking specific aspects of hypoxic adaptation could potentially reproduce the cardioprotective phenotype without the need for physical hypoxic exposure. The therapeutic potential of small-molecule inhibitors targeting m^6^A regulators has already been demonstrated *in vitro* and in animal models (An and Duan, 2022). Notably, STC-15 – an inhibitor of the m^6^A writer METTL3 – has recently become the first RNA-modifying enzyme inhibitor to enter clinical trials in cancer treatment (NCT05584111) (Medicine, 2025), bringing the concept of therapeutic epitranscriptomic manipulation closer to clinical application. A precise understanding of the role of m^6^A regulation in cardioprotection is therefore of vital importance for the rational design of such strategies.

## 5 Conclusion

In summary, our integrative multi-omics analysis reveals that chronic normobaric hypoxia induces a coordinated remodeling of cardiac metabolism, protein homeostasis, and epitranscriptomic regulation consistent with a cardioprotective phenotype. Pathway enrichment analyses identified adaptive reprogramming of energy metabolism (AMPK, glycolysis, and PPAR signaling), reinforcement of antioxidant defenses (glutathione metabolism), and structural remodeling of lipid membranes (glycerophospholipid and peroxisomal pathways), accompanied by activation of protein quality control mechanisms (autophagy–lysosome and proteasome systems). Beyond these canonical adaptations, we demonstrate for the first time that chronic hypoxia profoundly alters the m^6^A/m^6^Am regulatory network, characterized by increased abundance of the demethylases ALKBH5 and FTO, upregulation of multiple YTH-domain m^6^A readers, and a selective increase in global m^6^Am levels despite stable m^6^A abundance. This epitranscriptomic remodeling may represent an additional, previously unrecognized layer of hypoxia-induced cardioprotection, linking metabolic flexibility with post-transcriptional control of gene expression. Together, these findings provide a system-level framework for understanding how prolonged hypoxic adaptation reprograms the heart toward enhanced tolerance to ischemic stress.

## Data Availability

The datasets presented in this study can be found in online repositories. The names of the repository/repositories and accession number(s) can be found in the article/Supplementary Material.

## References

[B1] Alanova P. Chytilová A. Neckář J. Hrdlička J. Míčová P. Holzerová K. (2017). Myocardial ischemic tolerance in rats subjected to endurance exercise training during adaptation to chronic hypoxia. J. Appl. Physiol. (1985) 122, 1452–1461. 10.1152/japplphysiol.00671.2016 28209739

[B2] Alanova P. Alan L. Opletalova B. Bohuslavova R. Abaffy P. Matejkova K. (2024). HIF-1α limits myocardial infarction by promoting mitophagy in mouse hearts adapted to chronic hypoxia. Acta Physiol. (Oxf) 240, e14202. 10.1111/apha.14202 39016532

[B3] An Y. Duan H. (2022). The role of m6A RNA methylation in cancer metabolism. Mol. Cancer 21, 14. 10.1186/s12943-022-01500-4 35022030 PMC8753874

[B4] Balkova P. Jezková J. Hlavácková M. Neckár J. Stanková B. Kolár F. (2009). Dietary polyunsaturated fatty acids and adaptation to chronic hypoxia alter acyl composition of serum and heart lipids. Br. J. Nutr. 102, 1297–1307. 10.1017/s0007114509389242 19480730

[B5] Benak D. Benakova S. Plecita-Hlavata L. Hlavackova M. (2023). The role of m6A and m6Am RNA modifications in the pathogenesis of diabetes mellitus. Front. Endocrinol. (Lausanne) 14, 1223583. 10.3389/fendo.2023.1223583 37484960 PMC10360938

[B6] Benak D. Kolar F. Hlavackova M. (2024a). Epitranscriptomic regulations in the heart. Physiol. Res. 73, S185–S198. 10.33549/physiolres.935265 38634649 PMC11412340

[B7] Benak D. Holzerova K. Hrdlicka J. Kolar F. Olsen M. Karelson M. (2024b). Epitranscriptomic regulation in fasting hearts: implications for cardiac health. RNA Biol. 21, 1–14. 10.1080/15476286.2024.2307732 38326277 PMC10854364

[B8] Benak D. Holzerova K. Kolar F. Chalupova M. Hlavackova M. (2024c). Unveiling the proteome of the fasting heart: insights into HIF-1 pathway regulation. Front. Physiol. 15, 1462014. 10.3389/fphys.2024.1462014 39469441 PMC11513464

[B9] Benak D. Sevcikova A. Holzerova K. Hlavackova M. (2024d). FTO in health and disease. Front. Cell Dev. Biol. 12, 1500394. 10.3389/fcell.2024.1500394 39744011 PMC11688314

[B10] Benak D. Alanova P. Holzerova K. Chalupova M. Opletalova B. Kolar F. (2025). Epitranscriptomic regulation of HIF-1: bidirectional regulatory pathways. Mol. Med. 31, 105. 10.1186/s10020-025-01149-x 40102715 PMC11917031

[B11] Benak D. Hlavackova M. Nossent A. Y. Sopić M. (2026). Transcriptomics in atherosclerosis. Elsevier. 113–128.

[B12] Cairns M. Joseph D. Essop M. F. (2022). The dual role of the hexosamine biosynthetic pathway in cardiac physiology and pathophysiology. Front. Endocrinol. (Lausanne) 13, 984342. 10.3389/fendo.2022.984342 36353238 PMC9637655

[B13] Cajka T. Smilowitz J. T. Fiehn O. (2017). Validating quantitative untargeted lipidomics across nine liquid chromatography-high-resolution mass spectrometry platforms. Anal. Chemistry 89, 12360–12368. 10.1021/acs.analchem.7b03404 29064229

[B14] Cajka T. Hricko J. Rudl Kulhava L. Paucova M. Novakova M. Kuda O. (2023). Optimization of Mobile phase modifiers for fast LC-MS-Based untargeted metabolomics and lipidomics. Int. Journal Molecular Sciences 24, 1987. 10.3390/ijms24031987 36768308 PMC9916776

[B15] Chavez J. C. Agani F. Pichiule P. LaManna J. C. (2000). Expression of hypoxia-inducible factor-1alpha in the brain of rats during chronic hypoxia. J. Appl. Physiol. (1985) 89, 1937–1942. 10.1152/jappl.2000.89.5.1937 11053346

[B16] Choi M. Chang C. Y. Clough T. Broudy D. Killeen T. MacLean B. (2014). MSstats: an R package for statistical analysis of quantitative mass spectrometry-based proteomic experiments. Bioinformatics 30, 2524–2526. 10.1093/bioinformatics/btu305 24794931

[B17] Chytilová A. Borchert G. H. Mandíková-Alánová P. Hlaváčková M. Kopkan L. Khan M. A. H. (2015). Tumour necrosis factor-α contributes to improved cardiac ischaemic tolerance in rats adapted to chronic continuous hypoxia. Acta Physiol. (Oxf) 214, 97–108. 10.1111/apha.12489 25760892

[B18] Deng W. Jin Q. Li L. (2021). Protective mechanism of demethylase fat mass and obesity-associated protein in energy metabolism disorder of hypoxia-reoxygenation-induced cardiomyocytes. Exp. Physiol. 106, 2423–2433. 10.1113/ep089901 34713923

[B19] Dontaine J. Bouali A. Daussin F. Bultot L. Vertommen D. Martin M. (2022). The intra-mitochondrial O-GlcNAcylation system rapidly modulates OXPHOS function and ROS release in the heart. Commun. Biol. 5, 349. 10.1038/s42003-022-03282-3 35414690 PMC9005719

[B20] Essop M. F. (2007). Cardiac metabolic adaptations in response to chronic hypoxia. J. Physiol. 584, 715–726. 10.1113/jphysiol.2007.143511 17761770 PMC2276994

[B21] Guo Z. Tian Y. Liu N. Chen Y. Chen X. Yuan G. (2024). Mitochondrial stress as a central player in the pathogenesis of hypoxia-related myocardial dysfunction: new insights. Int. J. Med. Sci. 21, 2502–2509. 10.7150/ijms.99359 39439461 PMC11492880

[B22] Hao Z. Wu T. Cui X. Zhu P. Tan C. Dou X. (2020). N(6)-Deoxyadenosine methylation in mammalian mitochondrial DNA. Mol. Cell. 78, 382–395. 10.1016/j.molcel.2020.02.018 32183942 PMC7214128

[B23] Hlavácková M. Neckár J. Jezková J. Balková P. Stanková B. Nováková O. (2007). Dietary polyunsaturated fatty acids alter myocardial protein kinase C expression and affect cardioprotection induced by chronic hypoxia. Exp. Biol. Med. (Maywood) 232, 823–832. 10.3181/00379727-232-2320823 17526775

[B24] Hlaváčková M. Kožichová K. Neckář J. Kolář F. Musters R. J. P. Novák F. (2010). Up-regulation and redistribution of protein kinase C-δ in chronically hypoxic heart. Mol. Cell Biochem. 345, 271–282. 10.1007/s11010-010-0581-8 20853175

[B25] Hlavackova M. Benak D. Holzerova K. Alanova P. Hrdlicka J. Chalupova M. (2025). Epitranscriptomic signatures in blood: emerging biomarkers for diagnosis of diabetes and its complications. Front. Cell Dev. Biol. 13, 1656769. 10.3389/fcell.2025.1656769 41195338 PMC12583049

[B26] Holzerova K. Hlaváčková M. Žurmanová J. Borchert G. Neckář J. Kolář F. (2015). Involvement of PKCepsilon in cardioprotection induced by adaptation to chronic continuous hypoxia. Physiol. Res. 64, 191–201. 10.33549/physiolres.932860 25317680

[B27] Hu L. Wang J. Huang H. Yu Y. Ding J. Yu Y. (2021). YTHDF1 regulates pulmonary hypertension through translational control of MAGED1. Am.J.Respir.Crit Care Med. 203, 1158–1172. 10.1164/rccm.202009-3419OC 33465322

[B28] Hurtado A. (1960). Some clinical aspects of life at high altitudes. Ann. Intern Med. 53, 247–258. 10.7326/0003-4819-53-2-247 14405552

[B29] Ingelson-Filpula W. A. Kadamani K. L. Ojaghi M. Pamenter M. E. Storey K. B. (2024). Hypoxia-induced downregulation of RNA m(6)A protein machinery in the naked mole-rat heart. Biochimie 225, 125–132. 10.1016/j.biochi.2024.05.017 38788827

[B30] Kadamani K. L. Rahnamaie-Tajadod R. Eaton L. Bengtsson J. Ojaghi M. Cheng H. (2024). What can naked mole-rats teach us about ameliorating hypoxia-related human diseases? Ann. N. Y. Acad. Sci. 1540, 104–120. 10.1111/nyas.15219 39269277

[B31] Kanehisa M. (2019). Toward understanding the origin and evolution of cellular organisms. Protein Sci. 28, 1947–1951. 10.1002/pro.3715 31441146 PMC6798127

[B32] Kanehisa M. Goto S. (2000). KEGG: kyoto encyclopedia of genes and genomes. Nucleic Acids Res. 28, 27–30. 10.1093/nar/28.1.27 10592173 PMC102409

[B33] Kanehisa M. Furumichi M. Sato Y. Matsuura Y. Ishiguro-Watanabe M. (2025). KEGG: biological systems database as a model of the real world. Nucleic Acids Res. 53, D672–d677. 10.1093/nar/gkae909 39417505 PMC11701520

[B34] Kohler D. Staniak M. Tsai T. H. Huang T. Shulman N. Bernhardt O. M. (2023). MSstats version 4.0: statistical analyses of quantitative mass spectrometry-based proteomic experiments with chromatography-based quantification at scale. J. Proteome Res. 22, 1466–1482. 10.1021/acs.jproteome.2c00834 37018319 PMC10629259

[B35] Kolar F. Ostadal B. (2004). Molecular mechanisms of cardiac protection by adaptation to chronic hypoxia. Physiol. Res. 53 (Suppl. 1), S3–S13. 10.33549/physiolres.930000.53.s3 15119931

[B36] Kumar S. Mohapatra T. (2021). Deciphering epitranscriptome: modification of mRNA bases provides a new perspective for post-transcriptional regulation of gene expression. Front. Cell Dev. Biol. 9, 628415. 10.3389/fcell.2021.628415 33816473 PMC8010680

[B37] Medicine N. L. (2025). Oral administration of STC-15 in subjects with advanced malignancies (NCT05584111). Available online at: https://clinicaltrials.gov/study/NCT05584111?term=NCT05584111&rank=1.

[B38] Micova P. Hahnova K. Hlavackova M. Elsnicova B. Chytilova A. Holzerova K. (2016). Chronic intermittent hypoxia affects the cytosolic phospholipase A(2)α/cyclooxygenase 2 pathway *via* β(2)-adrenoceptor-mediated ERK/p38 stimulation. Mol. Cell Biochem. 423, 151–163. 10.1007/s11010-016-2833-8 27686454

[B39] Moritz C. P. (2017). Tubulin or not tubulin: heading toward total protein staining as loading control in Western blots. Proteomics 17, 1600189. 10.1002/pmic.201600189 28941183

[B40] Neckar J. Svatoňová A. Weissová R. Drahota Z. Zajíčková P. Brabcová I. (2017). Selective replacement of mitochondrial DNA increases the cardioprotective effect of chronic continuous hypoxia in spontaneously hypertensive rats. Clin. Sci. (Lond) 131, 865–881. 10.1042/cs20170083 28292971

[B41] Oerum S. Meynier V. Catala M. Tisné C. (2021). A comprehensive review of m6A/m6Am RNA methyltransferase structures. Nucleic Acids Res. 49, 7239–7255. 10.1093/nar/gkab378 34023900 PMC8287941

[B42] Qiu Z. Cui J. Huang Q. Qi B. Xia Z. (2024). Roles of O-GlcNAcylation in mitochondrial homeostasis and cardiovascular diseases. Antioxidants (Basel) 13, 571. 10.3390/antiox13050571 38790676 PMC11117601

[B43] Rohart F. Gautier B. Singh A. Lê Cao K. A. (2017). mixOmics: an R package for 'omics feature selection and multiple data integration. PLoS Comput. Biol. 13, e1005752. 10.1371/journal.pcbi.1005752 29099853 PMC5687754

[B44] Seagroves T. N. Ryan H. E. Lu H. Wouters B. G. Knapp M. Thibault P. (2001). Transcription factor HIF-1 is a necessary mediator of the pasteur effect in mammalian cells. Mol. Cell Biol. 21, 3436–3444. 10.1128/mcb.21.10.3436-3444.2001 11313469 PMC100265

[B45] Semenovykh D. Benak D. Holzerova K. Cerna B. Telensky P. Vavrikova T. (2022). Myocardial m6A regulators in postnatal development: effect of sex. Physiol. Res. 71, 877–882. 10.33549/physiolres.934970 36426889 PMC9814979

[B46] Shi D. L. (2023). RNA-binding proteins as critical post-transcriptional regulators of cardiac regeneration. Int. J. Mol. Sci. 24, 12004. 10.3390/ijms241512004 37569379 PMC10418649

[B47] Shi Y. Fan S. Wu M. Zuo Z. Li X. Jiang L. (2019). YTHDF1 links hypoxia adaptation and non-small cell lung cancer progression. Nat. Commun. 10, 4892. 10.1038/s41467-019-12801-6 31653849 PMC6814821

[B48] Singh A. Shannon C. P. Gautier B. Rohart F. Vacher M. Tebbutt S. J. (2019). DIABLO: an integrative approach for identifying key molecular drivers from multi-omics assays. Bioinformatics 35, 3055–3062. 10.1093/bioinformatics/bty1054 30657866 PMC6735831

[B49] Sistilli G. Kalendova V. Cajka T. Irodenko I. Bardova K. Oseeva M. (2021). Krill oil supplementation reduces exacerbated hepatic steatosis induced by thermoneutral housing in mice with diet-induced obesity. Nutrients 13, 437. 10.3390/nu13020437 33572810 PMC7912192

[B50] Song H. Feng X. Zhang H. Luo Y. Huang J. Lin M. (2019). METTL3 and ALKBH5 oppositely regulate m(6)A modification of TFEB mRNA, which dictates the fate of hypoxia/reoxygenation-treated cardiomyocytes. Autophagy 15, 1419–1437. 10.1080/15548627.2019.1586246 30870073 PMC6613905

[B51] Su Y. Xu R. Zhang R. Qu Y. Zuo W. Ji Z. (2020). N6-methyladenosine methyltransferase plays a role in hypoxic preconditioning partially through the interaction with lncRNA H19. Acta Biochim. Biophys. Sin. (Shanghai) 52, 1306–1315. 10.1093/abbs/gmaa130 33197240

[B52] Tanabe A. Tanikawa K. Tsunetomi M. Takai K. Ikeda H. Konno J. (2016). RNA helicase YTHDC2 promotes cancer metastasis *via* the enhancement of the efficiency by which HIF-1a mRNA is translated. Cancer Lett. 376, 34–42. 10.1016/j.canlet.2016.02.022 26996300

[B53] Thalhammer A. Bencokova Z. Poole R. Loenarz C. Adam J. O'Flaherty L. (2011). Human AlkB homologue 5 is a nuclear 2-oxoglutarate dependent oxygenase and a direct target of hypoxia-inducible factor 1a (HIF-1a). PLoS.One. 6, e16210. 10.1371/journal.pone.0016210 21264265 PMC3021549

[B54] Tsugawa H. Ikeda K. Takahashi M. Satoh A. Mori Y. Uchino H. (2020). A lipidome atlas in MS-DIAL 4. Nat. Biotechnology 38, 1159–1163. 10.1038/s41587-020-0531-2 32541957

[B55] Wang Y. J. Yang B. Lai Q. Shi J. F. Peng J. Y. Zhang Y. (2021). Reprogramming of m(6)A epitranscriptome is crucial for shaping of transcriptome and proteome in response to hypoxia. RNA.Biol. 18, 131–143. 10.1080/15476286.2020.1804697 32746693 PMC7834094

[B56] Wang J. Li Y. Deng L. Zha Y. Zhang S. (2024). FTO suppresses cardiac fibrosis after myocardial infarction *via* m(6)A-mediated epigenetic modification of EPRS. Mol. Med. 30, 213. 10.1186/s10020-024-00985-7 39538146 PMC11562098

[B57] Xu Z. Qin Y. Lv B. Tian Z. Zhang B. (2022). Intermittent fasting improves high-fat diet-induced obesity cardiomyopathy *via* alleviating lipid deposition and apoptosis and decreasing m6A methylation in the heart. Nutrients 14, 251. 10.3390/nu14020251 35057432 PMC8781965

[B58] Yao M. D. Jiang Q. Ma Y. Liu C. Zhu C. Y. Sun Y. N. (2020). Role of METTL3-Dependent N(6)-Methyladenosine mRNA modification in the promotion of angiogenesis. Mol. Ther. 28, 2191–2202. 10.1016/j.ymthe.2020.07.022 32755566 PMC7545007

[B59] Yuan S. Schmidt H. M. Wood K. C. Straub A. C. (2021). CoenzymeQ in cellular redox regulation and clinical heart failure. Free Radic. Biol. Med. 167, 321–334. 10.1016/j.freeradbiomed.2021.03.011 33753238 PMC12882998

[B60] Zhao K. Yang C. Zhang J. Sun W. Zhou B. Kong X. (2021). METTL3 improves cardiomyocyte proliferation upon myocardial infarction *via* upregulating miR-17-3p in a DGCR8-dependent manner. Cell Death.Discov. 7, 291. 10.1038/s41420-021-00688-6 34645805 PMC8514505

