# Supplementary material for: Integrative multi-omic profiling of the chronically hypoxic heart: focus on m6A and m6Am epitranscriptomic regulation
Source: Front Cell Dev Biol. 2026 Jan 28;14:1756287. doi: 10.3389/fcell.2026.1756287 (PMC12891189; doi:10.3389/fcell.2026.1756287)
Supplement: Supplementary file 9 [file DataSheet1.docx]

**Supplementary material**

**
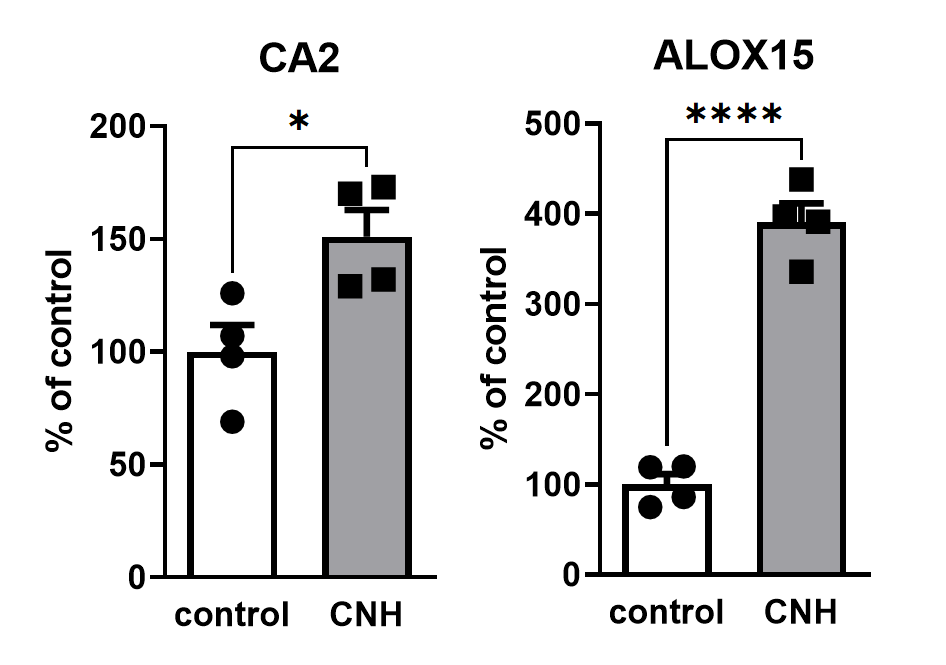
**

**Fig. S1**: Confirmation of proteomic results by Western blot. Antibodies: anti-CA2 (Abcam, UK; ab124687, 1:1,000, overnight), anti-ALOX15 (Abcam, UK; ab244205, 1:1,000, overnight), -and anti-rabbit (Bio-Rad, USA; 170-6515, 1:10,000, 1 h), the loadings were 5 ug (CA2) and 20 ug (ALOX15). n = 4; * p < 0.05, **** p < 0.0001 :(t-test). ALOX15 – 15 lipoxygenase 1; CA2 – carbonic anhydrase 2.

**
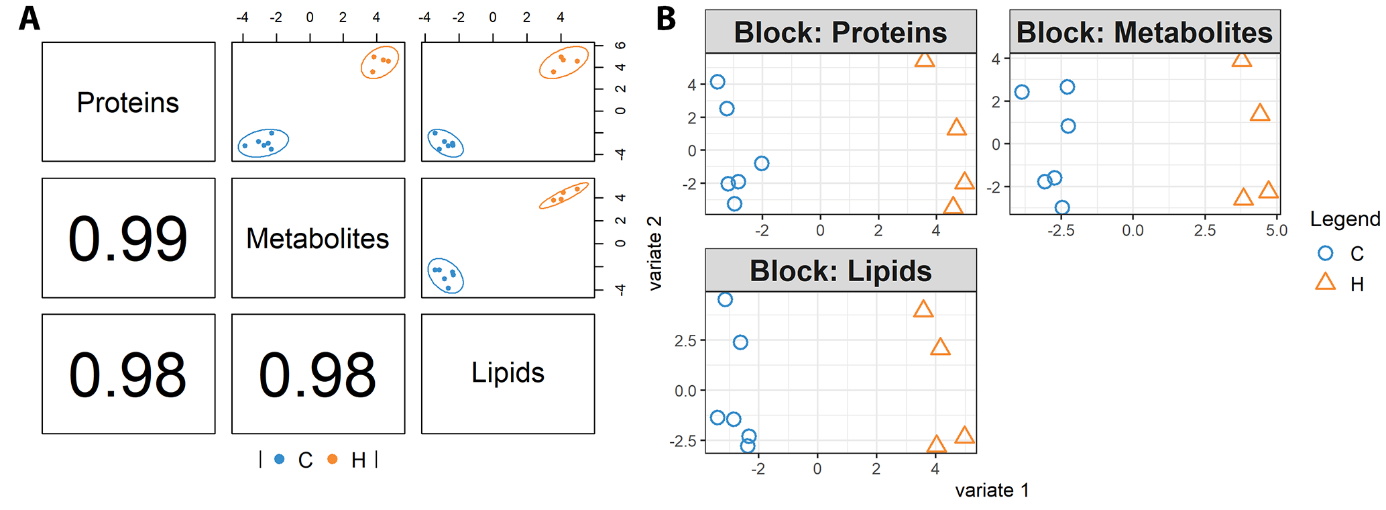
**

**Fig. S2:** Diagnostic plots as an initial step of multi-omics DIABLO integration. (A) Plot from multiblock PLS-DA shows that experimental groups are discriminated by component 1 and indicates strong correlations between first components from each data set. Correlation coefficients are shown on the bottom left. (B) Samples plotted according to their PLS-DA scores on the components (variates) 1 and 2 for each omics set.


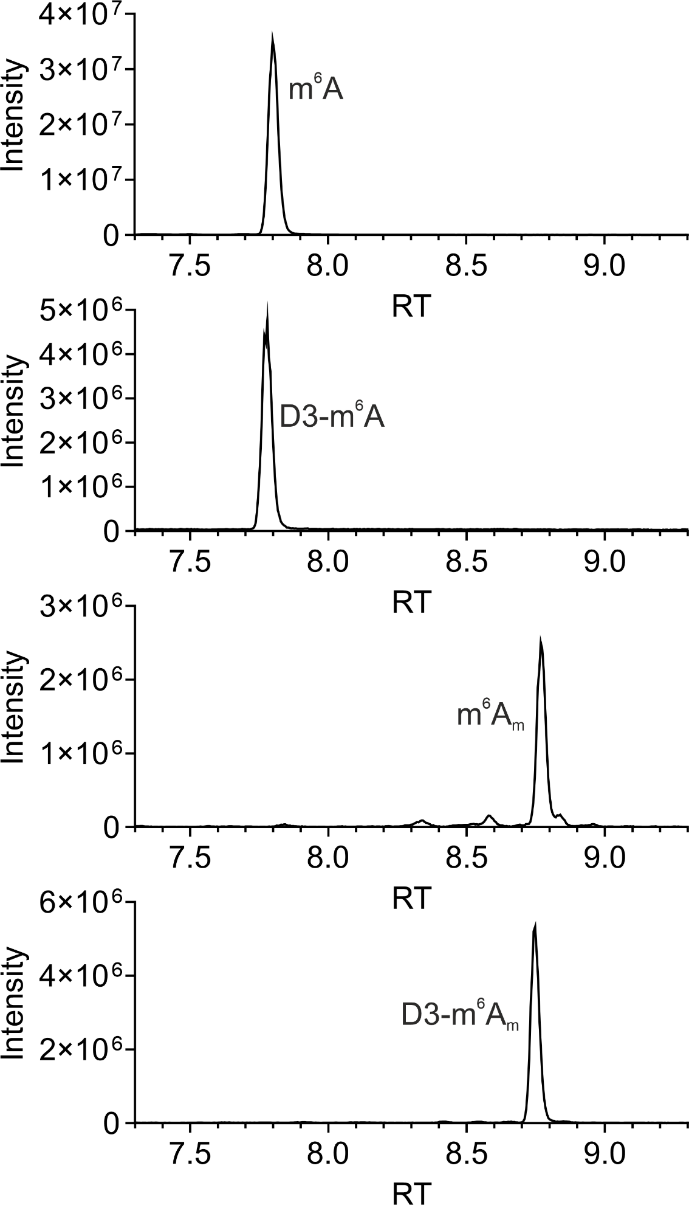


**Fig. S3:** Extracted ion chromatograms of modified nucleosides m^6^A and m^6^A_m_ from digested RNA and isotopically labeled standards D3-m^6^A and D3-m^6^A_m_ used for quantification.
